# Modified first-level reconstruction and reinforcement during laparoscopic total hysterectomy for prevention of post-operative pelvic organ prolapse: a randomized clinical trial

**DOI:** 10.4314/ahs.v24i4.15

**Published:** 2024-12

**Authors:** Xiangru Chen, Hongbo Gao

**Affiliations:** Dazhu People's Hospital Affiliated to North Sichuan Medical College, 635100 Dazhu, China

**Keywords:** laparoscopy, total hysterectomy, vaginal stump, pelvic floor dysfunction

## Abstract

**Objective:**

This paper investigated that the clinical value of modified first-level reconstruction reinforcement in the prevention and treatment of pelvic floor dysfunction after laparoscopic total hysterectomy.

**Methods:**

A total of 360 patients undergoing laparoscopic total hysterectomy from December 2018 to September 2021 were selected and divided into three groups (A, B, C) according to POP-Q criteria: This is a randomized clinical trial in which women with first- and second-degree pelvic organ prolapse, and women without pelvic organ prolapse were each randomized into 3 arms of the study. According to the informed consent of patients, three groups are as following: Arm 1: 60 cases in the non-suspension group (vaginal stump was only sutured continuous absorbable suture); Arm 2: 60 cases in traditional suspension group (as in Arm 1, plus suspension of vaginal stump with non-absorbable sutures to cardinal and round ligaments); Arm 3: 60 cases in the modified suspension group (vaginal stump reinforced with horizontal reconstruction). POP-Q score, sexual life quality questionnaire, urinary incontinence questionnaire and pelvic floor ultrasound were compared before and at 6 and 12 months after operation.

**Results:**

(1) in the non-prolapse group and the prolapse group, the POP-Q score of the modified suspension group c was superior to that of the non-suspension group and the traditional suspension group b at 6 and 12 months after surgery (P < 0.05), and the postoperative POP-Q score of the prolapse group was significantly improved compared with that before surgery. (2) In both the non-prolapse and the prolapse study groups, the participants that were randomized to the modified suspension treatment (arm 3) had significantly better sexual function scores than those in arm 1 and arm 2 six and twelve months after surgery (p <0.05). (3) Similarly, participants in both the non-prolapse and the prolapse groups that were randomized to the modified suspension treatment arm (arm 3) were significantly less prone to. urinary incontinence than those randomized to arm 1 and arm 2 at 6 and 12 months after surgery.

**Conclusions:**

Compared with traditional vaginal stump suture and traditional vaginal stump suspension, the modified first-level reconstruction and consolidation method can effectively prevent and cure pelvic floor dysfunction after laparoscopic total hysterectomy, and significantly improve the quality of life of patients.

## Introduction

With the concept of minimally invasive deeply rooted in the hearts of the people, laparoscopic hysterectomy has been widely carried out and has become the third largest gynecological operation 1,2,3. Many studies have pointed out that pelvic floor dysfunction such as pelvic organ prolapse, sexual dysfunction and stress urinary incontinence may be secondary to total hysterectomy 4,5,6,7, the incidence of pelvic floor dysfunction after laparoscopic total hysterectomy is close to 50%^7^. When it comes clinical work, some patients undergoing laparoscopic hysterectomy have asymptomatic pelvic organ prolapse of degree II and below. For this type of patients, there is no standard or guideline for the treatment of pelvic organ prolapse at the same time. According to the clinical background investigation of this study, postoperative pelvic organ prolapse was aggravated in this kind of patients. Therefore, in view of the above two problems, the purpose of this study was to explore 1) the preventive effect of modified first-level reconstruction after laparoscopic total hysterectomy on pelvic floor dysfunction after laparoscopic total hysterectomy; 2) the therapeutic effect of modified first-level reconstruction in patients with asymptomatic first- and second-degree pelvic organ prolapse.

## Materials and methods

### General information

360 patients from December 2018 to September 2021, who underwent laparoscopic total hysterectomy due to benign diseases in Dazhu County people's Hospital were selected. According to the POP-Q score standard 10, the patients were divided into two groups: non-prolapse group and prolapse group, 180 cases in the non-prolapse group were not associated with pelvic organ prolapse, and 180 cases in the prolapse group were associated with asymptomatic first- and second-degree pelvic organ prolapse. The non-prolapse group and the prolapse group were randomly divided into three arms, which are non-suspension arm (arm 1) (n = 60), traditional suspension arm (arm 2) (n = 60) and modified suspension arm (arm 3) (n = 60).

### Mode of operation

(1) Perform laparoscopic total hysterectomy according to routine steps; (2) Arm 1 (the non-suspension arm): continuous suture of the vaginal stump with absorbable suture; Arm 2 (the traditional suspension arm): the vaginal stump was sutured with conventional absorbable sutures, and the non-absorbable sutures were reversed in a “C” shape to fix the sacral main ligament, round ligament, and vaginal stump angle on both sides. Arm 3 (the modified suspension arm): modified I horizontal reconstruction and reinforcement method was used to suture the vaginal stump: Before dealing with the blood vessels on both sides of the uterus, the course of the ureter was identified, the posterior leaf of the broad ligament was fully opened, and the lateral side of the sacral ligament about 1.5 cm on the side of the vaginal stump was freed to avoid the injury of the ureter during the posterior suture. After the vaginal stump was sutured with an absorbable suture, the two uterosacral ligaments on both sides were paralleled to the vaginal stump side with a non-absorbable suture, and two ligaments were sutured to form a ridge. The distance between the two needles was 1.5 cm. The needle passed through the posterior vaginal wall between the sutured uterosacral ligaments on both sides, but did not penetrate the mucosa. The cardinal ligament, round ligament and anterior vaginal wall (without penetrating the mucosa) were fixed on the sacral spine with non-absorbable suture, and the main ligament, round ligament and anterior vaginal wall were pulled moderately.

### Observation index

POP-Q score of each point in the pelvis: the higher the value, the lower the location of pelvic organs^8^;

Female Sexual Function Questionnaire: score the score ranges from 2 to 95, the higher the score, the better the performance;

**Pelvic floor muscle strength test score:** the patient contracts the vagina under the guidance of the doctor, and the doctor's finger is placed on the patient's vagina to feel the vaginal contraction, with a score of 0-5, 0 point: Fingers do not perceive vaginal muscle contractions, 1 point: Fingers detect vaginal muscle tremors; 2 points: Incomplete contraction of vaginal muscles, able to repeat 2 times for 2 seconds each; 3 points: Vaginal muscles contract completely and can be repeated 3 times for 3 seconds each time, but there is no confrontation; 4 points: Vaginal muscle contraction completely, can be repeated 4 times, 4 seconds each time, can slightly resist, 5 points: The vaginal muscles are completely contracted and can be repeated 5 times for a duration of more than 5 seconds, and there is continuous confrontation. The higher the score, the better the pelvic floor muscle strength; A score below 3 indicates a lack of pelvic floor muscle strength;

International consultation on incontinence questionnaire-overactive bladder questionnaire (ICIQ-O1.3 Observation indicators AB) score 9, the score ranges from 0 to 16. The higher the score, the greater the chance of overactive bladder and urinary incontinence; Compare the indexes of the corresponding groups 6 months and 12 months after operation.

### Statistical analysis

SPSS statistical software was used. The measurement data were expressed as (Mean ± SD), and ANOVA analysis was used for comparison between groups. When *P* < 0.05, LSD test was used for multiple 0.05. Enumeration data were compared using Fisher exact test, *P* < 0.05 was considered statistically significant.

## Result

In both the prolapse and non-prolapse groups, participants in the 3 comparative study groups (A, B, and C) had comparable age, parity, BMI, menopausal status, surgical bleeding volume, hospitalization days, indwelling catheter days, and incident vaginal stump infection (*P* >0.05). as shown in Table 1.

**Table 1:** Basic situation

Group		N	Age (years)	Number of births (times)	BMI	Menopausal status (cases/%)	Days of hospitalization (days)	Intraoperative bleeding volume)	Indwelling catheter days (days)	Anal exhaust time (days)	Vaginal stump infection (case / %)
Nonprolapse group	A	60	47.50±3.91	2.28±0.58	21.13±2.14	7(11.7%)	8.07±0.92	48.25±3.84	1.57±0.96	1.60±0.91	1 (1.67%)
	B	60	48.15±3.01	2.27±0.66	20.34±1.45	9(15.0%)	8.15±0.88	47.83±4.15	1.68±0.85	1.50±0.89	0
	C	60	48.63±4.59	2.15±0.61	22.01±2.05	10(16.7%)	8.00±0.86	47.33±4.46	1.72±1.08	1.68±0.11	0
	F/*χ*^2^		1.282	0.83	5.60	0.629	0.43	0.705	0.40	0.53	2.01
	P		0.280	0.438	0.382	0.730	0.65	0.495	0.67	0.59	0.68
Prolapse group	A	60	48.90±2.59	2.87±1.31	23.34±1.23	9(15.0%)	7.78±1.40	41.42±13,63	1.88±1.34	1.90±1.27	0
	B	60	48.78±2.64	2.78±1.30	22.78±2.16	13(21.7%)	7.73±1.546	38.67±15.12	1.58±1.17	1.60±0.98	1(1.67%)
	C	60	48.78±2.64	2.47±1.16	22.69±1.67	12(20.0%)	8.12±1.47	40.92±13.42	1.65±1.12	1.62±0.92	0
	F/*χ*^2^		1.04	1.69	3.76	0.943	1.25	0.65	1.01	1.50	1.01
	P		0.96	0.19	0.875	0.624	0.29	0.52	0.37	0.23	0.60

In the non-prolapse group and the prolapse group, there was no significant difference in the preoperative POP-Q scores of the non-suspension group, the traditional suspension group, and the modified suspension group, and they were comparable. See Table 2.

**Table 2:** POP-Q score before operation in non-prolapse group and prolapse group (Mean ± SD)

Group		N	Aa	Ba	C	D	Ap	Bp
Non-prolapse group (N=180)	a	60	-3±0	-3±0	-5.50±0.39	-7.50±0.39	-3±0	-3±0
	b	60	-3±0	-3±0	-5.38±0.39	-7.61±0.49	-3±0	-3±0
	c	60	-3±0	-3±0	-5.35±0.37	-7.38±0.39	-3±0	-3±0
	*F*				2.50	2.50		
	*P*				0.085	0.085		
	a	60	-0.94±0.58	-0.42±0.51	-2.34±0.49	-4.34±0.49	-1.00±0.62	-0.85±0.77
	b	60	-0.98±0.64	-0.55±0.53	-2.30±0.55	-4.30±0.55	-0.98±0.67	-0.81±0.78
Prolapse group (N=180)	*c*	60	-0.84±0.76	-0.63±0.74	-2.28±0.59	-4.27±0.59	-0.91±0.73	-0.78±0.84
	*F*		0.724	1.971	0.231	0.231	0.316	0.135
	*P*		0.486	0.142	0.794	0.794	0.730	0.874

### Comparison of POP-Q scores in non-prolapse group and prolapse group at 6 months and 1 year after operation

In the non-prolapse group and the prolapse group, the POP-Q value of c modified suspension group was better than a non-suspension group and b traditional suspension group at 6 months and 12 months after operation, and the differences were statistically significant (P<0.05), see Tables 3 and 4.

**Table 3:** Comparison of POP-Q at 6 months and 12 months after operation in non-prolapse group (Mean ± SD)

Time	Group	N	Aa	Ba	C	Ap	Bp
6 months after surgery	a	60	-2.39±0.51^c^	-2.26±0.52^c^	-4.69±0.74^c^	-2.52±0.35^bc^	-2.13±0.68^bc^
	b	60	-2.53±0.48^c^	-2.47±0.46^c^	-4.83±0.68^c^	-2.70±0.39^a^	-2.60±0.37^a^
	c	60	-2.72±0.35^ab^	-2.78±0.37^ab^	-6.63±0.82^ab^	-2.78±0.35^a^	-2.78±0.35^a^
	*F*		8.21	19.1	124.67	7.86	24.14
	*P*		<0.0001	<0.0001	<0.0001	<0.0001	<0.0001
	a	60	--2.29±0.47^c^	-2.01±0.62^c^	-4.57±0.46^c^	-2.21±0.42^c^	-2.08±0.55^c^
12 months after surgery	b	60	-2.33±0.49^c^	-2.10±0.68^c^	-4.68±0.56^c^	-2.30±0.44^c^	-2.17±0.56^c^
	c	60	-2.68±0.29^ab^	-2.55±0.39^ab^	-6.55±0.84^ab^	-2.59±0.46^ab^	-2.54±0.49^ab^
	*F*		14.50	15.26	180.91	12.33	12.46
	*P*		<0.0001	<0.0001	<0.0001	<0.0001	<0.0001

Note: The significance level of the average difference was 0.05.

**Table 4:** Comparison of POP-Q values in the prolapse group at 6 months and 12 months after operation (Mean ± SD)

Time	Group	N	Aa	Ba	C	Ap	Bp
	a	60	-0.67±0.56^c^	-0.01±0.71^bc^	-2.07±0.61^c^	-0.55±0.67^c^	-0.51±0.81^c^
6 months after surgery	b	60	-0.93±0.60	-0.51±0.55^a^	-2.13±0.65^c^	-0.87±0.74	-0.81±0.78
	c	60	-1.08±0.98^a^	-0.73±0.90^a^	-4.32±0.57^ab^	-0.97±0.75^a^	-0.96±0.81^a^
	*F*		4.78	14.91	263.13	5.55	4.94
	*P*		0.009	<0.0001	<0.0001	0.005	0.008
	a	60	-0.38±0.71^bc^	0.13±0.74^bc^	-1.73±0.84^c^	-0.21±0.80^bc^	-0.01±0.83^bc^
12 months after surgery	b	60	-0.81±0.62^a^	-0.43±0.68^a^	-1.84±0.83^c^	-0.81±0.77^a^	-0.72±0.87^a^
	c	60	-1.00±1.01^a^	-0.68±0.92	-4.24±0.63^ab^	-0.87±0.76^a^	-0.84±0.83^a^
	*F*		9.65	16.57	202.73	13.29	17.94
	*P*		<0.0001	<0.0001	<0.0001	<0.0001	<0.0001

Note: The significance level of the average difference was 0.05.

Comparison of the POP-Q score of different study arms In the non-prolapsed group, the POP-Q values Aa, Ba, Ap, and Bp in the c-modified suspension group were slightly lower than those before surgery at 6 months and 12 months after surgery, and the differences were statistically significant (*P* < 0.05). The POP-Q values of point C at 6 months and 12 months after operation were better than those before operation, and the difference was statistically significant (*P* < 0.05). The variance analysis showed that there was no significant difference between the scores of 12 months and 6 months after operation, as shown in table 5.

**Table 5:** Comparison of POP-Q values of non-prolapse group c modified suspension group before operation, 6 months and 12 months after operation (Mean ± SD)

Group	N	Aa	Ba	C	D	Ap	Bp
Before operation	60	-3±0^bc^	-3±0^bc^	-5.35±0.38^bc^	-7.35±0.37	-3±0^bc^	-3±0^bc^
Six months after operation	60	-2.73±0.35^a^	-2.78±0.37^ac^	-6.63±0.82^a^		-2.78±0.35^ac^	-2.74±0.40^ac^
12 months after operation	60	-2.68±0.30^a^	-2.55±0.39^ab^	-6.65±0.84^a^		-2.59±0.46^ab^	-2.54±0.49^ab^
*F*		26.82	31.51	60.90		22.81	23.97
*P*		<0.0001	<0.0001	<0.0001		<0.0001	<0.0001

In the prolapse group, the POP-Q values of Aa, Ba, Ap, and Bp in the c-modified suspension group were slightly lower than those before surgery at 6 months and 12 months after surgery. The difference was not statistically significant (*P* > 0.05), The POP-Q values of point C at 6 months and 12 months after operation were better than those before operation, and the difference was statistically significant (*P* < 0.05). Multiple comparisons after variance analysis showed that the POP-Q values of point C at 6 months and 12 months after operation were significantly different from those before operation. There was no significant difference in POP-Q values between 12 months after operation and 6 months after operation, as shown in Table 6.

**Table 6:** Comparison of POP-Q values in prolapse group c modified suspension group before operation, 6 months and 12 months after operation (Mean ± SD)

Group	N	Aa	Ba	C	D	Ap	Bp
Before operation	60	-0.84±0.76	-0.63±0.74	-2.28±0.59^bc^	-4.28±0.59	-0.91±0.73	-0.78±0.83
Six months after operation	60	-1.07±0.97	-0.73±0.90	-4.32±0.57^a^		-0.97±0.75	-0.96±0.81
12 months after operation	60	-1.00±1.00	-0.68±0.93	-4.24±0.63^a^		-0.87±0.76	-0.84±0.83
F		1.00	0.172	227.96		0.273	0.761
P		0.369	0.842	<0.0001		0.761	0.469

Note: The significance level of the average difference was 0.05.

There was no difference in the preoperative female sexual function questionnaire score, pelvic floor muscle strength test score and ICIQ-OAB score between the non-prolapse group and the prolapse group in a non-suspension group, b traditional suspension group, and c modified suspension group Statistical significance (*P* > 0.05). The scores of female sexual function questionnaire in the modified suspension group c were higher than those in the control group a and the control group b at 6 months and 12 months after the operation, and the difference was statistically significant (*P* < 0.05); The scores of pelvic floor muscle strength test in the modified suspension group were higher than those in a non-suspension group and b traditional suspension group at 6 months and 12 months after operation, and the difference was statistically significant (*P* < 0.05); c modified suspension group The ICIQ-OAB scores of the suspension group c were lower than those of the a non-suspension group and the b traditional suspension group at 6 months and 12 months after the operation, and the difference was statistically significant (*P* < 0.05), as shown in Tables 7 and 8.

**Table 7:** Comparison of sexual function questionnaire score, pelvic floor muscle strength test score, ICIQ-OAB score in non-prolapse group before operation, 6 months and 12 months after operation (Mean ± SD)

Time	Group	N	Sexual Function Questionnaire Score	Pelvic floor muscle strength test score	ICIQ-OAB score
	a	60	55.20±3.86	4.05±0.91	2.67±1.54
Before operation	b	60	55.13±3.83	4.08±0.91	2.67±1.60
	c	60	56.20±3.55	4.03±0.88	2.72±1.54
	*F*		1.525	0.048	0.020
	*P*		0.220	0.953	0.980
	a	60	50.35±3.94^c^	3.45±1.03^c^	4.75±0.27^c^
Six months after operation	b	60	50.57±34.37^c^	3.85±1.10^a^	4.15±1.88^c^
	c	60	56.07±3.78^ab^	3.97±0.79^c^	2.73±1.78^ab^
	*F*		38.623	4.575	14.477
	*P*		<0.0001	0.012	<0.0001
	a	60	46.65±3.87^c^	3.13±0.16^c^	5.07±2.64^c^
1 year after operation	b	60	46.23±3.42^c^	3.55±1.19	4.65±2.21^c^
	c	60	55.7±3.99^ab^	3.83±0.94^c^	2.93±1.82^ab^
	*F*		121.028	6.149	15.204
	*P*		<0.0001	0.003	<0.0001

Note: The significance level of the average difference was 0.05.

**Table 8:** Comparison of sexual function questionnaire scores, pelvic floor muscle strength test scores, ICIQ-OAB scores in the prolapse group before surgery, 6 months after surgery, and 12 months after surgery (Mean ± SD)

Time	Group	N	Sexual Function Questionnaire Score	Pelvic floor muscle strength test score	ICIQ-OAB score
	a	60	47.88±3.77	3.13±1.19	2.58±1.41
Before operation	b	60	47.68±3.66	3.11±1.21	2.60±1.42
	c	60	47.62±3.59	2.97±1.10	2..63±1.54
	*F*		0.086	0.506	0.018
	*P*		0.918	0.690	0.793
	a	60	42.77±3.49^c^	2.87±1.20^c^	2.90±1.35^c^
Six months after operation	b	60	42.78±3.54^c^	2.98±1.31^c^	2.77±1.35^c^
	c	60	50.86±3.13^ab^	3.62±1.17^ab^	2.18±1.47^ab^
	*F*		113.85	6.50	4.497
	*P*		<0.0001	0.002	0.012
	a	60	41.30±3.32^c^	2.43±1.21^c^	3.05±1.35^c^
1 year after operation	b	60	41.37±3.31^c^	2.50±1.28^c^	3.00±0.41^c^
	c	60	51.87±4.16^ab^	3.03±1.35^ab^	2.33±1.54^ab^
	*F*		169.486	3.938	4.668
	*P*		<0.0001	0.021	0.011

Note: The significance level of the average difference was 0.05.

## Discussion

Through the quantitative comparison of the degree of pelvic organ prolapse before and after operation by POP-Q score, it was found that the measured values of Aa, Ba, C, Ap and Bp in c modified suspension group at 6 and 12 months after operation were significantly higher than those in no suspension group a and traditional suspension group b, suggesting that modified I horizontal reconstruction and reinforcement can prevent pelvic organ prolapse after laparoscopic total hysterectomy to some extent. Compared with the traditional vaginal stump suspension, modified-I horizontal reconstruction and reinforcement are more effective in preventing pelvic organ prolapse after total hysterectomy. However, Aa, Ba, Ap, and Bp points of modified-I horizontal reconstruction and reinforcement were slightly lower than those before operation, and point C was significantly higher than that before operation, indicating that the improved first-level reconstruction and reinforcement mainly acts on the top of the vagina, has a significant effect on preventing vaginal vault prolapse, and has a significant effect on preventing uterine vault prolapse. The anterior and posterior vaginal wall bulging effect was not obvious after total resection. However, the scores of sexual function questionnaire and pelvic floor muscle strength test in modified suspension group c were significantly higher than those in no suspension group a and suspension group b, and the ICIQ-OAB score in modified suspension group was significantly lower than that in no suspension group a and traditional suspension group b.

According to Delancey's theory of “three levels of vaginal support”, the fascia and ligaments supporting the vagina are divided into three levels: first (upper), second (middle) and third (lower). The first-level is the top support, which is composed of the cervical fascia ring and the principal sacral ligament complex, and which vertically supports the upper 1/3 of the uterus and vagina and is the main support force of the pelvic floor. The second-level is that the pubic cervical fascia is attached to the bilateral pelvic fascia tendon arch to form the white line and the rectovaginal fascia anal levator muscle midline. The second-level can support the bladder, lower vaginal 2/3 and rectum. The third-level is the distal supporting structure, dominated by the perineal body and part of the levator ani muscle. After laparoscopic total hysterectomy, the cervical fascial annulus and the main sacral ligament complex in the supportive connective tissue of the first-level were severed 12-13. A number of studies have pointed out that after laparoscopic hysterectomy, the main sacral ligament-vaginal ring reconstruction can replace the supporting role of the cervical ring and can prevent pelvic floor dysfunction after total hysterectomy to a certain extent 14,15,16, Therefore, the reinforcement and reconstruction of first-level after total hysterectomy is necessary, clinically feasible, and has a certain curative effect. The modified-I horizontal reconstruction and reinforcement method in this study is to suture the vaginal stump in the low suspension sacral ligament while appropriately lifting the vaginal wall, main ligament and round ligament.

It is suggested that the incidence of decreased pelvic floor muscle strength, subjective bladder discomfort, urinary incontinence, decreased sexual function and pelvic organ prolapse after laparoscopic total hysterectomy is lower than that of traditional vaginal stump suture and traditional vaginal stump suspension. it is helpful to improve the quality of life of patients after total hysterectomy. The reason may be that the modified first-level reconstruction and reinforcement can fix the top of the vagina at a higher position and maintain the length of the vagina, so it can have a better quality of sexual life; and the tough sacral ligament formed in the modified I-level reconstruction and reinforcement. The ridge can fix the cardinal ligament, the anterior vaginal wall, the posterior vaginal wall, the bladder and the rectum well, maintain the normal anatomical position of the pelvic organs except the uterus and appendages, and reduce the probability of overactive bladder, so urinary incontinence and The incidence of bladder discomfort is low, the pressure of pelvic floor support second- and third- level after surgery does not increase significantly, and the pressure on pelvic floor muscles does not increase significantly compared with preoperative pressure, so the incidence of decreased pelvic floor muscle strength is low 17. All the patients in the group did not feel obvious lumbosacral pain, which was considered because the modified first-level reconstruction and reinforcement only shortened the upper end of the sacral ligament and round ligament of the cervical segment, pulled the main ligament and the anterior and posterior wall of the vagina, and after the release of laparoscopic pneumoperitoneum, the tension was significantly reduced, which was not enough to pull the sympathetic nerve of the cervical sacral ligament and cause pain. All the enrolled patients did not complain about the defecation function being affected after the operation. It is considered that because the pelvic autonomic nerve outside the sacral ligament is mainly located below the deep uterine vein, the total hysterectomy and suspension of the cervical sacral ligament did not damage the pelvic autonomic nerve. The patient's bowel function did not change significantly after operation 18-23.

The pelvic organ prolapse treatment guidelines point out that the surgical indication of pelvic organ prolapse is symptomatic pelvic organ prolapse 24. For patients with asymptomatic pelvic organ prolapse who need laparoscopic total hysterectomy, there are no norms and guidelines for the simultaneous treatment of pelvic organ prolapse during laparoscopic total hysterectomy. According to the clinical background investigation of this project, pelvic organ prolapse was further aggravated in this kind of patients after total hysterectomy. A study by Ma Yuan et al pointed out that reconstruction of the sacral main ligament-vaginal ring after total hysterectomy can replace the pelvic floor support of the cervical ring to treat stage I pelvic organ prolapse and improve the quality of life of patients 25, However, the operation steps are more complicated, and an ultrasonic knife is required. The sacral ligament is sutured along the pelvic side wall of the vaginal stump. The actual operation space is limited, and the risk of ureteral injury is high. The modified-I horizontal reconstruction and reinforcement method for suturing the vaginal stump is to identify the shape of the ureter before dealing with the blood vessels on both sides of the uterus, fully open the posterior lobe of the broad ligament, dissociate the lateral sacral ligament of the vaginal stump about the lateral side of the 1.5cm, avoid injury to the ureter during posterior suture, and properly lift the vaginal wall, main ligament and round ligament at the same time of low suspended sacral ligament. For patients with pelvic organ prolapse, the measurements of Aa, Ba, C, Ap, and Bp points in the C modified suspension group in June and December were higher than those in the a-no suspension group and the b traditional suspension group. The C point was significantly improved compared with the preoperative period. The difference was significant, suggesting that the modified I horizontal reconstruction reinforcement surgery could treat asymptomatic I. and II. degree apical pelvic organ prolapses.

## Limitations

The current research is limited by the short follow-up period, and the small sample size. The quality of the study can be improved through multi-center randomized controlled clinical trials with larger sample sizes so as to provide more dependable results for evidence-based medical care.

## Conclusions

To sum up, after laparoscopic total hysterectomy, the modified first-level reconstruction and reinforcement method reconstructs and reinforces the disconnected vaginal supporting first-level connective tissue, and reduces postoperative pelvic floor muscle strength, subjective symptoms of bladder discomfort, urinary incontinence, and urinary incontinence. The incidence of sexual function decline and pelvic organ prolapse decrease, and it does not increase postoperative pain, and has no significant effect on defecation function. For patients with asymptomatic grade-I and grade-II pelvic organ prolapse, the treatment of pelvic organ prolapse to a certain extent improves the quality of life of patients after surgery. This operation is performed at the same time as total hysterectomy, which can reduce the medical burden caused by pelvic floor dysfunction after total hysterectomy, save medical resources, and save national medical insurance funds. It can also be used for abdominal hysterectomy. After short-term observation, the short-term curative effect is certain, and it has certain clinical value, which is worthy of promotion.
